# Accelerated Direct Identification of Gram-Positive and Gram-Negative Bacteria Using Matrix-Assisted Laser Desorption Ionization-Time of Flight Mass Spectrometry (MALDI-TOF MS) in Critically Ill Patients

**DOI:** 10.7759/cureus.108560

**Published:** 2026-05-09

**Authors:** Binal Mangroliya, Minakshi Singh, Vanya Singh, Ankit Agarwal, Mukesh Bairwa, Salabh Jauhari, Hitendra Singh, Pratima Gupta, Balram Omar

**Affiliations:** 1 Microbiology, All India Institute of Medical Sciences, Rishikesh, Rishikesh, IND; 2 Anaesthesiology, All India Institute of Medical Sciences, Rishikesh, Rishikesh, IND; 3 Internal Medicine, All India Institute of Medical Sciences, Rishikesh, Rishikesh, IND; 4 Microbiology, Government Doon Medical College, Dehradun, IND

**Keywords:** bloodstream infections, direct identification, maldi-tof ms, rapid diagnostics, turnaround time

## Abstract

Background: Bloodstream infections (BSIs) are associated with high morbidity and mortality, particularly in critically ill patients. Delayed bacterial identification may result in inappropriate empirical therapy and poor outcomes. Direct identification from positive blood culture bottles can significantly reduce turnaround time (TAT). While direct matrix-assisted laser desorption ionization-time of flight mass spectrometry (MALDI-TOF MS) has been evaluated for gram-negative bacilli (GNB), data on gram-positive cocci (GPC) remain limited. This study assessed direct MALDI-TOF MS performance for both GPC and GNB to support timely sepsis management.

Objective: The objective of the study was to compare direct bacterial identification by MALDI-TOF MS with the standard method of overnight incubation and processing using MALDI-TOF MS and to evaluate the reduction in TAT.

Methods: This prospective study was conducted from September 2024 to September 2025 at the Department of Microbiology of a tertiary care centre in North India. Adult ICU patients with two or more blood culture bottles flagged positive by BACT/ALERT® 3D (bioMérieux SA, Marcy-l'Étoile, France) and showing monomicrobial morphology on Gram stain (yeast excluded) were included, yielding 251 isolates. Direct MALDI-TOF MS was performed from positive broth; the standard method involved subculture on blood agar and MacConkey agar with 18-24 hours aerobic incubation at 37°C, followed by MALDI-TOF MS. Concordance was assessed using categorical agreement and Cohen's kappa.

Results: Among 251 monomicrobial isolates, 103 (41.0%) were GPC and 148 (59.0%) were GNB by the standard method. Direct MALDI-TOF MS achieved species-level identification in 82.5% of GPC and 85.1% of GNB isolates. Genus-level agreement was 96.1% for GPC and 85.8% for GNB; species-level agreement was 82.5% and 83.8%, respectively. Overall concordance was 84.1%, with almost perfect agreement (Cohen's κ = 0.82). Mean TAT decreased by 23.82 hours, with the Wilcoxon signed-rank test confirming a highly significant reduction (p < 0.001).

Discussion: Identification accuracy was slightly higher for GNB compared with GPC. The higher misidentification rate observed among GPC isolates may reflect the spectral similarity among closely related species. Significant reduction in TAT demonstrates the potential for earlier targeted antimicrobial therapy and improved clinical decision-making in critically ill patients.

Conclusion: Direct MALDI-TOF MS from positive blood cultures demonstrated high concordance with the standard method while significantly reducing TAT. The approach performed robustly for both GPC and GNB. This accelerated workflow can facilitate the timely optimization of antimicrobial therapy, strengthen antimicrobial stewardship, and may improve clinical outcomes in critically ill patients.

## Introduction

The World Sepsis Day theme of 2025, “5 Facts × 5 Actions,” highlighted five essential truths about sepsis and five urgent actions required to address it, emphasizing that most deaths from sepsis are preventable [1]. Bloodstream infections (BSIs) are one of the primary causes of morbidity and mortality globally, particularly among critically ill patients admitted to intensive care units (ICUs). Early and correct diagnosis is crucial for timely and appropriate management to prevent progression to sepsis [2]. According to estimates from the Global Burden of Disease Study, as referenced by the World Health Organization (WHO) and Global Sepsis Alliance, sepsis affects nearly 49 million people annually and contributes to approximately 11 million deaths worldwide, accounting for nearly 20% of all deaths [1,3,4]. These estimates highlight the substantial global burden of sepsis and the need for rapid diagnostic strategies.

Delayed management of sepsis may lead to complications such as septic shock, a subset of sepsis characterized by profound circulatory and cellular or metabolic abnormalities associated with significantly increased mortality [5]. Studies have shown that mortality increases by approximately 7.6% with every hour of delay in initiating effective antimicrobial therapy. Additionally, discordant empirical antibiotic therapy, particularly in infections caused by drug-resistant pathogens, is associated with increased mortality [6]. Therefore, early pathogen identification is critical for guiding targeted therapy, improving clinical outcomes, and reducing healthcare costs [7].

Blood culture remains the gold standard for diagnosing bloodstream infections. However, the standard workflow requires subculture of positive blood culture broth onto solid media followed by overnight incubation before identification using matrix-assisted laser desorption ionization-time of flight mass spectrometry (MALDI-TOF MS). This process typically requires 48 hours or more, delaying definitive results and potentially prolonging empirical antibiotic therapy [8-10].

MALDI-TOF MS has transformed microbial identification by enabling rapid and accurate species-level identification based on protein profiling. Direct identification of pathogens from positively flagged blood culture broths using MALDI-TOF MS has emerged as a promising strategy to significantly reduce turnaround time (TAT) and facilitate earlier organism-directed therapy. Rapid identification within hours of blood culture positivity can improve clinical decision-making and patient outcomes in patients with bacteraemia [11,12].

Most previous studies evaluating direct MALDI-TOF MS identification from positive blood cultures have predominantly focused on gram-negative bacilli (GNB), with limited data available for gram-positive cocci (GPC) [13,14]. Furthermore, some studies have utilized complex or commercially optimized extraction methods such as Sepsityper® (Bruker Corporation, Billerica, Massachusetts, United States), which may not be readily available in resource-limited settings [13,15]. These limitations highlight the need for evaluating simpler, cost-effective protocols applicable to routine clinical laboratories.

Therefore, this study aimed to compare direct MALDI-TOF MS identification of both GPC and GNB with the standard method of overnight subculture-based identification using MALDI-TOF MS and to evaluate the reduction in TAT.

## Materials and methods

Study design and setting

This prospective study was conducted from September 2024 to September 2025 in the Department of Microbiology, All India Institute of Medical Sciences (AIIMS), Rishikesh, a tertiary care centre in Uttarakhand, India. The study was approved by the Institutional Ethics Committee of AIIMS (Ref No. AIIMS/IEC/24/396).

Study population and sample size

Adult ICU patients from whom two or more blood culture bottles were received and flagged positive by BACT/ALERT® 3D (bioMérieux SA, Marcy-l'Étoile, France) blood culture system, and showed monomicrobial morphology on Gram stain (excluding yeast) were included, yielding 251 isolates for analysis. Sample size was determined based on the number of eligible positive blood culture samples obtained during the study period. A consecutive sampling strategy was followed, wherein all blood culture bottles meeting the inclusion criteria during the study period were included. Blinding was not performed, as both direct and standard identification methods were conducted within the same laboratory workflow; however, identification results were interpreted based on instrument-generated outputs, minimizing observer bias.

Blood culture processing

All blood culture bottles were incubated in the BacT/ALERT 3D blood culture system. The system detects microbial growth by measuring carbon dioxide (CO₂) production using a colorimetric sensor at the base that changes colour from blue-green to yellow as CO₂ accumulates during microbial metabolism. A sustained change in reflectance is interpreted as a positive signal. Blood culture bottles showing no colour change after seven days were reported as negative [16].

Direct microscopy

When a blood culture bottle was flagged positive, Gram staining was performed. A drop of broth from the positive bottle was aseptically placed on a clean glass slide, spread to form a thin smear, air-dried, and heat-fixed before Gram staining. Blood culture bottles showing more than one morphotype or the presence of yeasts on Gram staining were excluded from the study [17]. Blood culture bottles showing monomicrobial morphology on Gram stain, either gram-positive or gram-negative, were processed further for identification. 

Standard identification method

A 100 µL of positive blood culture broth was inoculated onto blood agar and MacConkey agar plates and incubated aerobically at 37°C for 18-24 hours. The resulting colonies were processed for identification using MALDI-TOF MS. A bacterial colony was smeared as a thin film onto a spot on the MALDI target plate. One microliter of matrix solution (α-cyano-4-hydroxycinnamic acid) was overlaid onto each spot and allowed to air dry. The target plate was then loaded into the MALDI-TOF MS system, and spectra were acquired for microbial identification using the instrument’s database [18,19]. Quality control for MALDI-TOF MS was performed as per manufacturer recommendations using standard calibration strains. Identification scores were interpreted according to the manufacturer’s criteria, with scores ≥2.0 considered reliable for species-level identification and scores between 1.7 and 1.99 considered acceptable for genus-level identification. Scores <1.7 were considered unreliable and reported as no identification.

Direct identification method

A 3 mL of positive blood culture broth was transferred into a serum separator tube (SST) and centrifuged at 3000 rpm for five minutes. The supernatant was discarded, and the volume was adjusted to 3 mL using sterile normal saline. The SST was centrifuged again at 3000 rpm for 15 minutes. After discarding the supernatant, the resulting pellet was used for microbial identification. The pellet was spotted onto the MALDI-TOF target plate and overlaid with 1 µL of matrix solution (α-cyano-4-hydroxycinnamic acid). After air drying, the target plate was inserted into the MALDI-TOF MS system for spectral acquisition and identification using the instrument database. 

Statistical analysis 

Statistical analysis was performed using IBM SPSS Statistics for Windows, version 26.0 (IBM Corp., Armonk, New York, United States). Concordance between direct and standard identification methods was assessed using categorical agreement and Cohen's kappa statistics. Symmetry of disagreement was evaluated using Bowker's test. Turnaround time between the two methods was compared using the Wilcoxon signed-rank test. A p-value of <0.05 was considered statistically significant.

## Results

A total of 251 monomicrobial isolates from 162 ICU patients with positive blood cultures were included in the analysis. The mean age of the study population was 46.98 ± 17.65 years, with a median age of 48 years. Among the patients, 88 (54.3%) were male, and 74 (45.7%) were female. Most blood culture samples were obtained from the Medicine ICU, followed by other specialty ICUs. Based on Gram stain morphology and identification by MALDI-TOF using the standard method, 103 isolates (41.04%) were GPC and 148 isolates (58.96%) were GNB.

**Figure 1:**
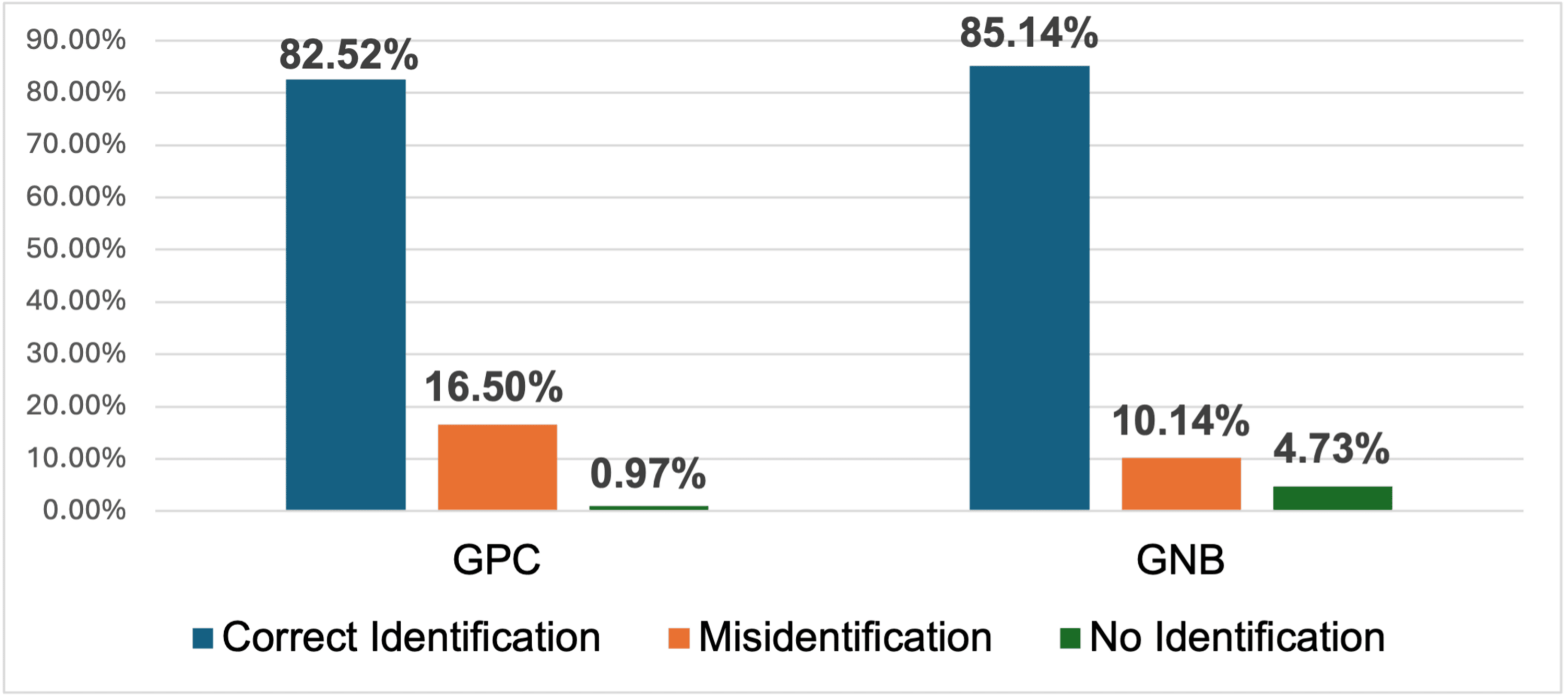
Comparison of identification outcomes for GPC and GNB GPC: gram-positive cocci; GNB: gram-negative bacilli

Direct identification by MALDI-TOF from positive blood culture broth was evaluated against the standard method. Among the 103 GPC isolates, correct identification was obtained in 85 isolates, while 17 were misidentified, and one remained unidentified. For the 148 GNB isolates, 126 isolates were correctly identified, 15 were misidentified, and seven yielded no identification (Figure 1).

**Figure 2:**
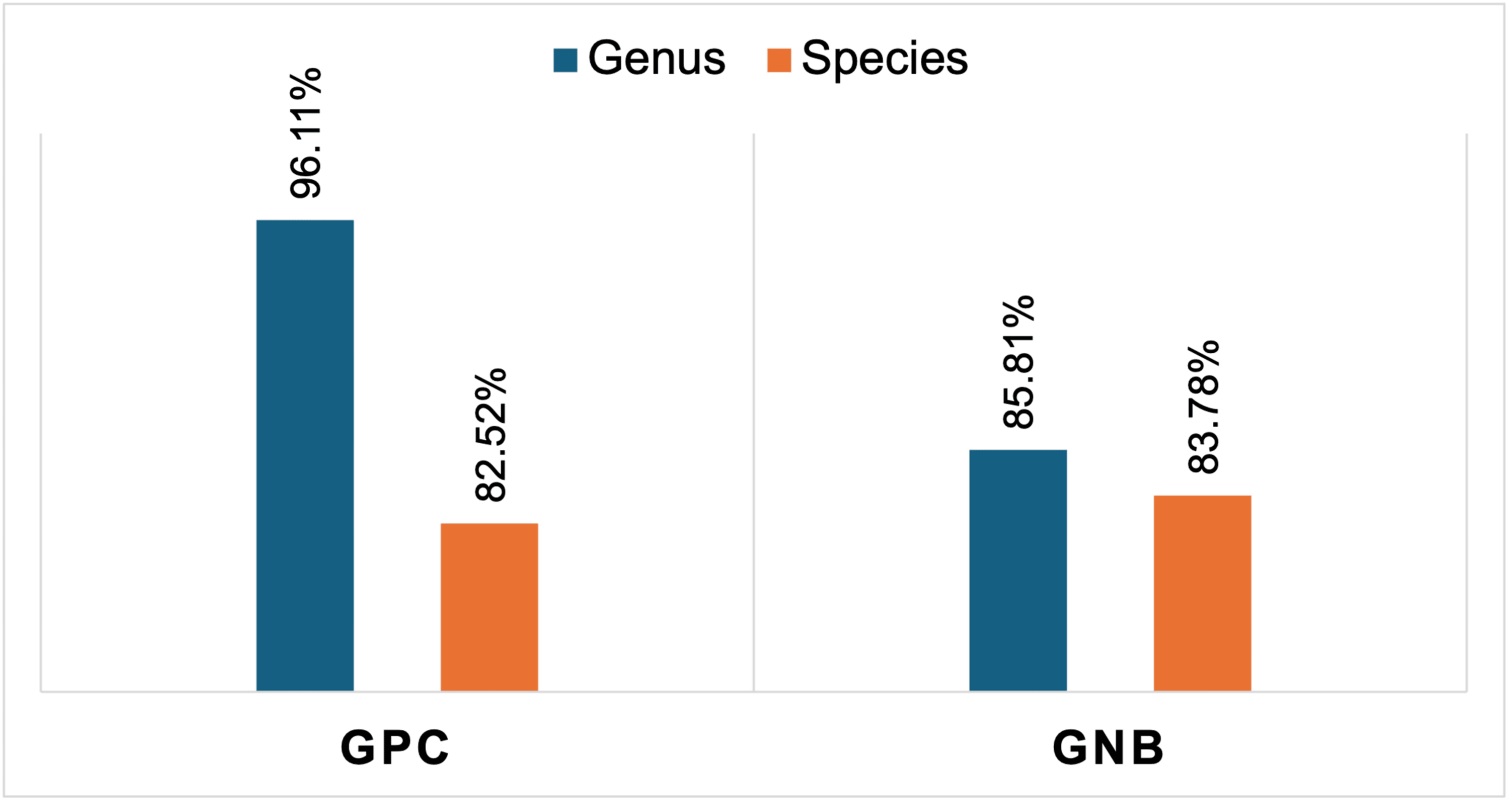
Genus and species level agreement in identification of GPC and GNB GPC: gram-positive cocci; GNB: gram-negative bacilli

When analysed according to taxonomic agreement, Genus-level agreement was higher than species-level agreement for GPC, whereas both levels were comparable for GNB (Figure 2). 

Statistical analysis demonstrated high concordance between direct and standard identification methods. For GPC isolates, the categorical agreement (CA) was 82.52%, with a Cohen’s kappa value of 0.74 (95% CI: 0.627-0.834), indicating substantial agreement. For GNB isolates, CA was 85.14%, with a Cohen’s kappa value of 0.82 (95% CI: 0.759-0.890), indicating almost perfect agreement. Bowker’s test showed no significant asymmetry for either group (p > 0.05). 

Overall, considering all isolates together, 211 of 251 isolates (84.06%) were correctly identified by the direct method. The overall Cohen’s kappa value was 0.82 (95% CI: 0.773-0.870), demonstrating strong agreement between direct and standard identification methods, with no evidence of systematic directional bias (Bowker χ² = 34.8, p = 1.000). Overall analysis of all 251 isolates demonstrated a significantly shorter turnaround time with the direct workflow compared with the standard method. The mean TAT decreased from 25.75 ± 0.06 hours to 1.93 ± 0.06 hours, representing an average reduction of 23.82 hours (92.5%). Statistical analysis using the Wilcoxon signed-rank test showed this reduction to be highly significant (p < 0.001) (Table 1).

**Table 1:** Comparison of TAT between direct and standard methods GPC: gram-positive cocci; GNB: gram-negative bacilli; TAT: turnaround time

Organism group	No. of isolates	Standard Method TAT (hours)	Direct Method TAT (hours)	Mean Reduction (hours)	% Reduction	p-value
GPC	103	25.75 ± 0.05	1.94 ± 0.05	23.81	92.5%	<0.001
GNB	148	25.75 ± 0.06	1.92 ± 0.06	23.83	92.5%	<0.001
Overall	251	25.75 ± 0.06	1.93 ± 0.06	23.82	92.5%	<0.001

Most misidentifications among GPC occurred within coagulase-negative *Staphylococcus* and *Enterococcus* species. Among GNB, misidentifications were predominantly observed in non-fermenting organisms, particularly involving *Pseudomonas aeruginosa*. Occasional intra-genus misidentifications were also observed within *Klebsiella* species and *Acinetobacter* species

## Discussion

The present study was conducted to evaluate the performance of direct microbial identification by MALDI-TOF MS from positive blood culture bottles. The results obtained from these rapid methods were compared with the standard workflow involving subculture followed by identification using MALDI-TOF MS. Rapid identification of pathogens from positive blood cultures is essential for the timely initiation of targeted antimicrobial therapy, which plays a critical role in improving outcomes in patients with bloodstream infections [20,21].

Previous studies using automated methods for direct Identification have mostly focused only on GNB [13,14]. In contrast, the present study evaluated the performance of direct identification using an automated system for both GPC and GNB, addressing an area in which available literature is relatively limited. Some studies have utilized VITEK® 2 (bioMérieux SA) for direct bacterial identification from positive blood cultures; however, VITEK 2 requires overnight subculture prior to processing, resulting in a longer turnaround time compared to direct MALDI-TOF MS identification [22,23]. 

For GPC, the correct identification rate of 82.52% obtained in the present study closely aligns with the 81.5% correct identification reported in the study by Lee et al. [24]. Similarly, Lin et al. reported a correct identification rate of 78.2% for gram-positive aerobes [25]. This supports the reproducibility of this approach. Minor variations across studies may reflect differences in sample preparation and extraction efficiency as well as the spectral similarity among gram-positive organisms. A study utilizing an in-house clot activator tube method across 186 samples reported genus-level and species-level agreement of 47.3% for GPC, which is lower than the agreement achieved in the present study [26]. This may suggest that extraction quality influences GPC identification performance.

Most misidentifications occurred within the coagulase-negative *staphylococci *(CoNS) group, particularly involving inter-species confusion between *Staphylococcus haemolyticus*, *Staphylococcus hominis*, *Staphylococcus warneri*, and *Staphylococcus lentus*, which share highly similar ribosomal protein profiles. Misidentification of *Enterococcus faecalis* as *Enterococcus faecium* reflects the well-recognized spectral similarity between these two species. Rare aberrant identifications, such as *S. haemolyticus *being misidentified as *Vibrio fluvialis*, are attributable to very low spectral scores leading to spurious database matches, underscoring the importance of correlating MALDI-TOF results with Gram stain findings.

For GNB, the present study demonstrated a correct identification rate of 85.14%, comparable to 85% reported using a similar two-stage centrifugation method [25], supporting the reliability of this approach. However, higher identification rates (95.2%) have been reported with optimized extraction protocols, such as the rapid Sepsityper kit [27]. This suggests that optimized extraction strategies can substantially enhance GNB identification. but may not be feasible in all laboratory settings. However, the added cost and complexity of such methods may limit their routine use in resource-limited settings.

Similarly, Loonen et al. reported 92% correct identification for gram-negative isolates using a centrifugation-washing protocol with absolute ethanol [28]. A study involving 96 gram-negative monomicrobial positive blood cultures demonstrated 94.7% correct identification, compared with 78.1% using the Sepsityper kit, emphasizing that sample processing has a substantial effect on identification yields [13]. Another study using an SST centrifugation method reported genus-level identification accuracy of 58.7% for GPC and 75.8% for GNB, while species-level accuracy was 26% and 75%, respectively [14]. These findings further support the observation that GNB generally demonstrate higher identification accuracy than GPC in direct MALDI-TOF workflows.

In the present study, misidentification patterns among GNB isolates were predominantly within non-fermenting organisms. These mainly involved *P. aeruginosa*, which was incorrectly identified as *Chryseobacterium indologenes*, *Sphingomonas paucimobilis*, and *Burkholderia cepacia* complex. This likely reflects overlapping low-score spectra under suboptimal direct extraction conditions despite phenotypic differences.

The present study also demonstrated a significant reduction in turnaround time with the direct workflow compared with the standard method. Overall analysis of 251 isolates showed that the mean TAT decreased from 25.75 ± 0.06 to 1.93 ± 0.06, representing an average reduction of 92.5%, which was statistically significant (p < 0.001). 

Overall, the findings support the utility of direct MALDI-TOF MS identification from positively flagged blood cultures as a rapid and reliable diagnostic approach. Earlier bacterial identification facilitates timely optimization of appropriate antimicrobial therapy in critically ill patients with bloodstream infections. In addition, prompt organism identification may aid in early source-directed management and infection control interventions, which are crucial in reducing morbidity and length of hospital stay.

Limitation

The present study is limited by its single-centre design. Larger, multicentric studies are required to validate and strengthen these observations. Additionally, polymicrobial blood cultures were excluded, which may limit the applicability of the findings to routine clinical scenarios. Identification accuracy is dependent on the quality and coverage of the MALDI-TOF MS database, which may influence performance for less common organisms.

Future prospects

Exploring the performance of direct methods across a wider spectrum of pathogens, including polymicrobial cultures and less commonly isolated organisms, may broaden their applicability. Emerging technologies such as biosensor-based platforms hold promise for further reducing diagnostic turnaround time in bloodstream infections and warrant evaluation in future studies.

## Conclusions

This study demonstrated that direct identification using MALDI-TOF MS from positive blood culture broth showed high concordance with standard culture-based methods while significantly reducing turnaround time. The earlier availability of identification may facilitate timely optimization of antimicrobial therapy in patients with bloodstream infections.

These findings support the incorporation of direct identification methods into routine laboratory practice as part of diagnostic stewardship initiatives. Such approaches may aid in earlier clinical decision-making, strengthen antimicrobial stewardship efforts, which may improve patient outcomes. Further multicentric studies with larger patient populations are needed to better establish the clinical and economic impact of rapid diagnostic methods in the management of bloodstream infections.
